# Identification and molecular characterization of bacteriophage phiAxp-2 of *Achromobacter xylosoxidans*

**DOI:** 10.1038/srep34300

**Published:** 2016-09-27

**Authors:** Erna Li, Zhe Yin, Yanyan Ma, Huan Li, Weishi Lin, Xiao Wei, Ruixiang Zhao, Aimin Jiang, Jing Yuan, Xiangna Zhao

**Affiliations:** 1 College of Food Science, South China Agricultural University, Guangzhou, 510642, China; 2 State Key Laboratory of Pathogen and Biosecurity, Beijing Institute of Microbiology and Epidemiology, Beijing, 100071, China; 3 College of Food Science, Henan Institute of Science and Technology, Xinxiang, 453003, China; 4 Institute of Disease Control and Prevention, Academy of Military Medical Sciences, Beijing, 100071, China

## Abstract

A novel *Achromobacter xylosoxidans* bacteriophage, phiAxp-2, was isolated from hospital sewage in China. The phage was morphologically and microbiologically characterized, and its one-step growth curve, host range, genomic sequence, and receptor were determined. Its morphology showed that phiAxp-2 belongs to the family *Siphoviridae*. Microbiological characterization demonstrated that pH 7 is most suitable for phage phiAxp-2; its titer decreased when the temperature exceeded 50 °C; phiAxp-2 is sensitive to ethanol and isopropanol; and the presence of calcium and magnesium ions is necessary to accelerate cell lysis and improve the formation of phiAxp-2 plaques. Genomic sequencing and a bioinformatic analysis showed that phage phiAxp-2 is a novel bacteriophage, consisting of a circular, double-stranded 62,220-bp DNA molecule with a GC content of 60.11% that encodes 86 putative open reading frames (ORFs). The lipopolysaccharide of *A. xylosoxidans* is involved in the adsorption of phiAxp-2.

*Achromobacter xylosoxidans* is an nonfermenting Gram-negative bacillus^1^. It is an uncommon opportunistic pathogen with low virulence, but can potentially cause invasive infections in immunocompromised patients, such as those with meningitis, empyema, pulmonary abscess, peritonitis, urinary tract infection, prosthetic valve endocarditis, chronic otitis media, keratitis, osteomyelitis, endophthalmitis, or septic arthritis^1^. *A. xylosoxidans* is frequently associated with antibiotic-resistant nosocomial infections. Bacteriophage therapy directed against *A. xylosoxidans* may be useful in combating these infections. Bacteriophages are potential therapeutic agents in the treatment of bacterial infections and useful diagnostic tools^2^, and since their discovery, attempts have been made to use bacteriophages to treat several infectious diseases^3^. Therefore, new phages are being isolated and characterized^4^. Because *A. xylosoxidans* infections are some of the most problematic nosocomial infections, the isolation and characterization of novel phages that infect this species is a priority. Many of these phages have been isolated in recent years, but only some have been fully sequenced and described in detail^5^. To develop an effective antimicrobial agent, we isolated a bacteriophage, designated phiAxp-2, from hospital sewage and described its morphology, host range, growth characteristics, whole genome sequence, and receptor usage. This phage may be an effective tool for the control of *A. xylosoxidans* infections in susceptible populations.

## Results and Discussion

### Phage morphology

**Figure 1:**
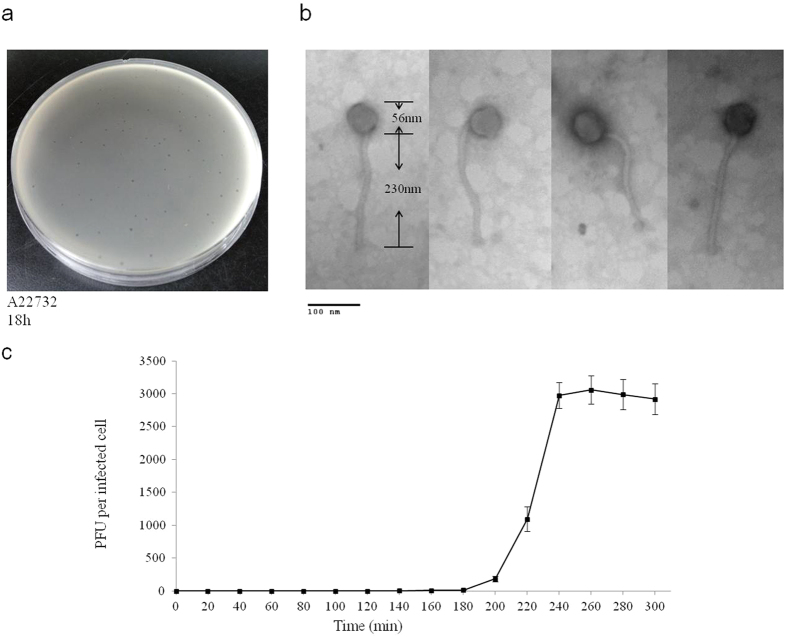
(**a**) Morphology of phiAxp-2 plaques. Phages were plated in Luria–Bertani agar and overlain with a liquid culture of *A. xylosoxidans* A22732. The plates were incubated at 37 °C. Clear, well-defined phiAxp-2 plaques were observed and photographed after 18 h. (**b**) Phage morphology. Phage was stained with 2% phosphotungstic acid and visualized at 120,000-fold magnification with transmission electron microscopy. Scale bars represent 100 nm. (**c**) One-step growth curve of the bacteriophage. The phage was grown in an exponential-phase culture of *A. xylosoxidans* strain A22732. Shown are the pfu per infected cell in the cultures at different time points. Each data point is the mean of three experiments.

Bacteriophage phiAxp-2 was isolated from *A. xylosoxidans* strain A22732 and observed with electron microscopy. *A. xylosoxidans* strain A22732 harbours a conjugative imipenem-encoding plasmid and is resistant to multiple β -lactam antibiotics, including imipenem and meropenem^6^.Cell lysis was observed after induction (phage yield: 1 × 10^9^ pfu/ml), by propagating the induced lysate on strain A22732. The plaques obtained had a clear pinpoint morphology, with well-defined boundaries (Fig. 1a). Electron micrographs of negatively stained phiAxp-2 virions showed an icosahedral head and a long noncontractile tail (Fig. 1b). The average particle had a capsid of approximately 56 nm in diameter and a tail length of approximately 230 nm, and the phage is therefore morphologically similar to phages in the order *Caudovirales* and family *Siphoviridae*. Host range tests suggested that among all the species tested (n = 14), phiAxp-2 was specifically virulent to only four strains of *A. xylosoxidans* (Table 1). Besides the reported multidrug-resistant strain A22732, the other three clinical *A. xylosoxidans* strains were shown to be resistant to aztreonam and tobramycin^6^.

### Latent period and burst size

There is a progressive relationship between burst size and the latent period, such that an optimal latent period leads to high phage fitness, and an increase in burst size may contribute to plaque size or larger plaques with higher burst sizes^7^,^8^. A one-step growth curve of phage phiAxp-2 propagated on *A. xylosoxidans* A22732 was constructed. The latent period of phage phiAxp-2 was 180 min. The burst time was approximately 240 min and the burst size was 2,985 pfu/cell (burst size = the total number of phages liberated at the end of one cycle of growth/number of infected bacteria) (Fig. 1c).

### Microbiological characterization

**Figure 2:**
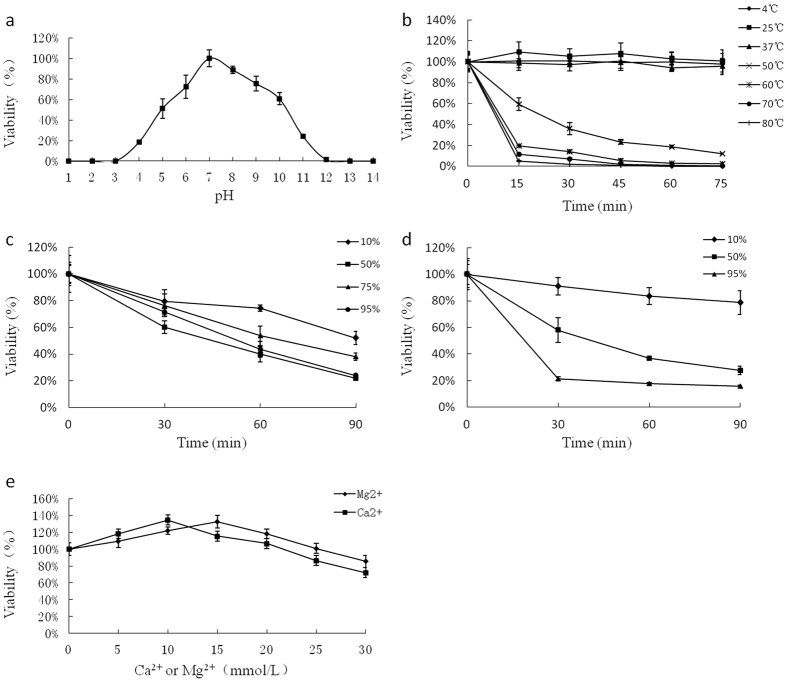
Resistance of phage phiAxp-2 to physical and chemical agents. (**a**) Effect of pH on the phage phiAxp-2 titer after incubation for 60 min in LB broth at 37 °C. (**b**) Inactivation kinetics of phage phiAxp-2 at 4 °C, 25 °C, 37 °C, 50 °C, 60 °C, 70 °C, and 80 °C. (**c**) Inactivation kinetics of phage phiAxp-2 in the presence of 10%, 50%, 75%, and 95% ethanol. (**d**) Inactivation kinetics of phage phiAxp-2 in the presence of 10%, 50%, and 95% isopropanol. (**e**) Viability of phage phiAxp-2 in LB broth with different Ca^2+^ and Mg^2+^ concentrations. On all graphs, the values are the means of three determinations.

Figure 2a shows the pH sensitivity of phage phiAxp-2. The phage maintained its infectivity when incubated at 37 °C in a pH range of 4–11. At pH 1 and pH 14, approximately 100% reductions in the phage particle counts were observed. The loss of viability when phage phiAxp-2 was subjected to various temperatures is shown in Fig. 2b. Phage phiAxp-2 displayed heat-sensitivity at 50 °C, 60 °C, 70 °C, and 80 °C. Treatment at 80 °C for 75 min completely inactivated the phage. As shown in Fig. 2c,d, the activity of phage phiAxp-2 was affected by the presence of ethanol and isopropanol. The most effective concentrations of ethanol (95%, v/v) and isopropanol (95%, v/v) reduced the phage titer by 76% and 84%, respectively, after 90 min. Because many phages require divalent ions (such as Ca^2+^ or Mg^2+^) for optimal adsorption^9^, the ion-dependence of phage phiAxp-2 was determined. The most efficient infection was achieved with concentrations of 15 mM Mg^2+^ and 10 mM Ca^2+^ (Fig. 2e).

### Genome characterization

**Figure 3:**
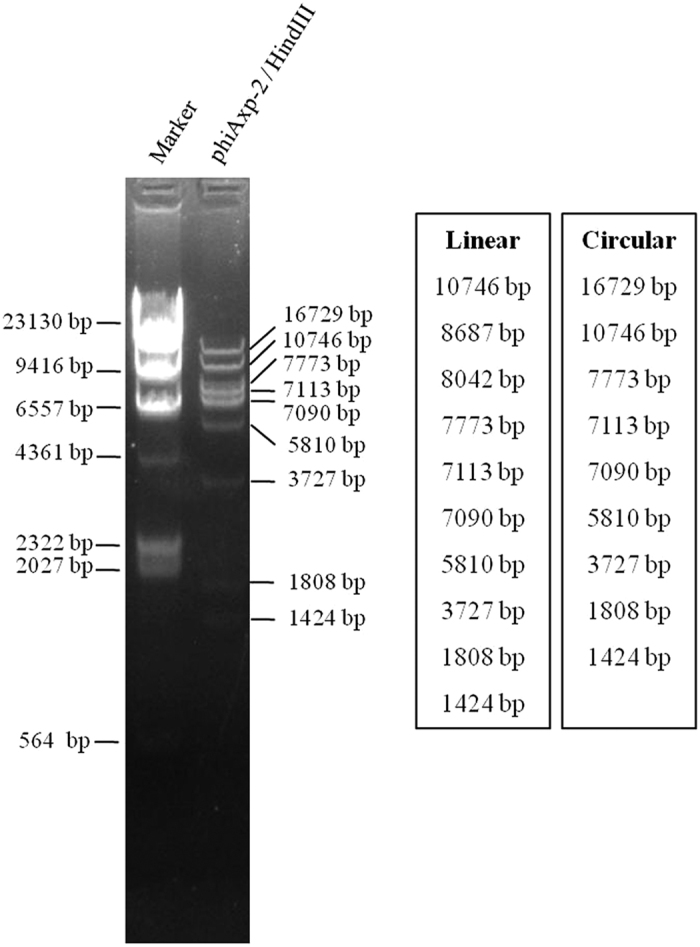
Restriction fragment length polymorphism analysis of phiAxp-2 DNA. Genomic DNA from phage phiAxp-2 was digested with the enzymes indicated (*HindIII*) and run on an agarose gel (0.7%). The length of fragments generated by digestion of the linear genome or the circular genome was showed on the right side of the electrophoresis map.

**Figure 4:**
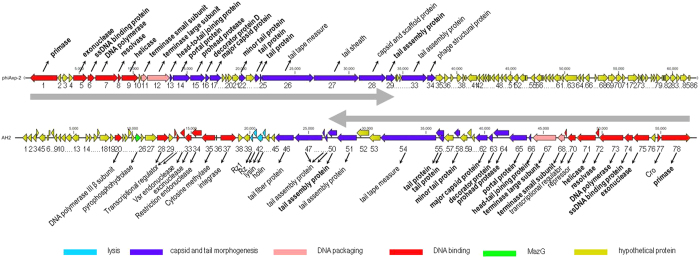
Genomic map of phiAxp-2. The genomic map of phiAxp-2 was constructed with CLC Main Workbench, version 6.1.1 (CLC bio, Aarhus, Denmark). The bacteriophage phiAxp-2 genome is schematically presented with the predicted ORFs indicated by arrows; the direction of the arrow represents the direction of transcription.

For the future application of phage phiAxp-2 to protect humans from *A. xylosoxidans* infections, the phage must be characterized in detail, including its genomic sequence. The genomic DNA of phiAxp-2 was extracted and purified, and its genome was completely sequenced and analyzed. Analysis of the sequence found that the restriction endonuclease *HindIII* had nine cutting sites in the genomic DNA. Thus, it was expected that when *HindIII* was used to digest the DNA, ten fragments would be generated if the DNA comprised a linear genome, but if the genome was circular, nine fragments would be generated. The *HindIII* digestion experiment generated nine fragments in the agarose gel (Fig. 3), revealing that the phiAxp-2 genome is a circular molecule. The complete circular double-stranded DNA genome of phage phiAxp-2 is 62,220 bp in length with a G+C content of 60.11%. This percentage is lower than those of the complete *Achromobacter* genomes sequenced so far (65–66% G+C content), but is higher than those of the sequenced *A. xylosoxidans*-specific phages, JWAlpha (KF787095) and JWDelta (KF787094), which are 54.4% and 54.2%, respectively^5^. Analysis of the phage phiAxp-2 genome revealed 86 putative open reading frames (ORFs). The National Center for Biotechnology Information (NCBI) database was scanned for homologues of the proteins encoded by the predicted ORFs using BlastP. Because the genome of phiAxp-2 diverges from other available phage genomes, only a limited number (33%) of protein functions could be predicted with similarity searches, highlighting the novelty of this phage. Therefore, a more detailed investigation is required to fully understand the nature of this novel phage. Twenty-eight ORFs showed some similarity in the BlastP analysis. Putative functions were assigned to 22 ORFs based on these similarities. Despite the low number of annotated ORFs, different modules can be recognized in the phiAxp-2 genome, which encodes proteins for (i) DNA replication, regulation, and modification, (ii) DNA packaging, and (iii) head and tail morphogenesis (Fig. 4). All of these functional clusters are located in ORF1–ORF34, which constitutes approximately 60% of the whole genome length. The remaining ORFs showed less or no similarity to other proteins in the NCBI database.

**Figure 5:**
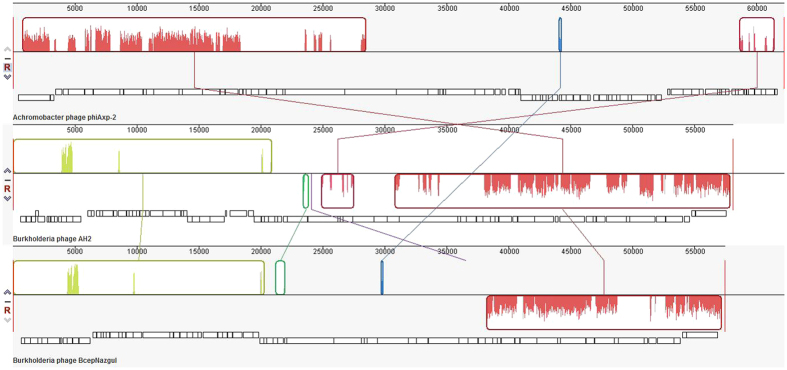
Multiple genome alignment constructed with the Mauve software (http://asap.ahabs.wisc.edu/mauve/) and the chromosomes of related phages. Similarity is represented by the heights of the bars, which correspond to the average level of conservation in that region of the genomic sequence. Completely white regions represent fragments that were not aligned or contained sequence elements specific to a particular genome. Boxes with identical colors represent local colinear blocks (LCB), indicating homologous DNA regions shared by two or more chromosomes without sequence rearrangements. LCBs indicated below the horizontal black line represent reverse complements of the reference LCB.

**Figure 6:**
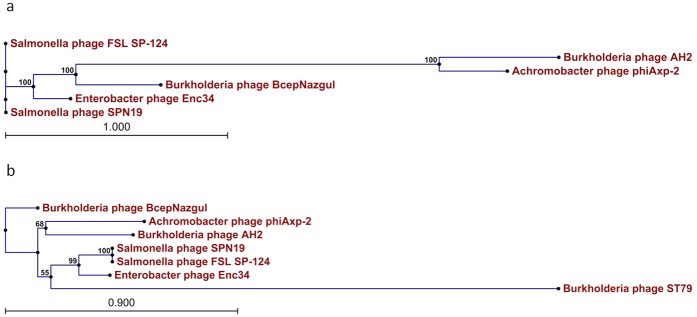
Phylogenetic tree based on DNA polymerases and the large terminase subunits of selected bacteriophages. The DNA polymerases and large terminase subunits were compared using the ClustalW program, and the phylogenetic tree was generated with the neighbor-joining method and 1000 bootstrap replicates (CLC Genomics Workbench 6).

The left half of the phiAxp-2 genome (ORF1–ORF29) has a similar genomic structure and encodes proteins most similar to each of the *Burkholderia* phage AH2 (JN564907) proteins from ORF78–ORF50 (the right half of the AH2 genome; excluding nine proteins) in the reverse transcription direction (Fig. 4 and Table 2). However, the range of overall similarity was as low as 29–57% at the amino acid level, and genomic comparisons with AH2 showed that in many instances, only small parts or domains of the ORFs were conserved. The most similar of these protein was the portal protein (57% identity with AH2 gp65) and the least similar was the decorator protein (24% identity with AH2 gp63) (Table 2). Phage AH2 is most closely related to *Burkholderia* phage BcepNazgul (NC005091)^10^, and a multiple genome alignment of the chromosomes of phiAxp-2, AH2, and BcepNazgul confirmed their relatedness (Fig. 5). Despite the similarities in their virion morphologies (the AH2 virion also has a noncontractile tail of approximately 220 nm and a capsid approximately 60 nm in diameter^10^), a phylogenetic analysis of the DNA polymerase and the terminase large subunit predicted that phiAxp-2 is most closely related to AH2 (Fig. 6a,b). Because the proteins of phiAxp-2 have largely uncharacterized functions, its genome must encode several new viral proteins. We predict that the large-scale genomic rearrangement in phiAxp-2 was mediated by transposase genes. However, we did not identify any transposase gene. Such genomic rearrangements may have caused the genomic diversity observed in the phages, resulting in the biological differences that distinguish them.

### Module analysis

Genes for DNA replication and DNA metabolism occur at the beginning of the phiAxp-2 genome, followed by packaging genes and the structural genes. ORF1–9 encode proteins for DNA replication, regulation, and modification, and six of them encode proteins with homology to AH2 proteins (35–45% identity): DNA primase (ORF1), exonuclease (ORF5), single-stranded DNA binding protein (ORF6), DNA polymerase (ORF7), resolvase (ORF8), and helicase (ORF9). The DNA packaging genes (the terminase subunits, ORF11–ORF12) of phiAxp-2 both have counterparts in AH2. phiAxp-2 ORF11 codes a 199-amino-acid (199-aa) protein with limited similarity (32%) to the known small terminase subunit of phage AH2. A DUF1441 superfamily member was detected using BlastP-based tools, and appears to be distantly related to other helix–turn–helix DNA-binding motif families, so may also be involved in the recognition of viral DNA and the subsequent initiation of viral packaging^11^. The gene immediately downstream from ORF11 encodes a 695-aa protein with 54% identity to the large terminase subunit of AH2, which implies a similar function in DNA packaging. ORF12 is predicted to have a GpA (pfam05876) domain, which is actively involved in the late stages of packaging, including DNA translocation. ORF13–ORF17 make up the capsid morphogenesis module, containing genes encoding the head–tail joining protein (ORF13), portal protein (ORF14), prohead protease (ORF15), decorator protein (ORF16), and major capsid protein (ORF17). Each of these proteins is similar to an AH2 protein, with percentage identities of 29–57% (Table 2). Among these proteins, the portal protein is thought to form the hole through which DNA is packaged into the prohead, and is also a part of the packaging motor^11^. All the genes encoded by ORF20–25 have counterparts in AH2 (31–55% identity), and three were annotated as tail proteins: ORF22, ORF24, and ORF25. Like most tailed phages, phiAxp-2 encodes two tail proteins proximal to the tail tape measure gene^12^. The tape measure protein of phiAxp-2, encoded by ORF26, has no sequence similarity to that of AH2, although the two proteins have similar functions in the assembly of the phage tails and in tail length determination^12^,^13^. phiAxp-2 ORF27 is distantly related to the *Escherichia* phage N4 tail sheath protein, which is known to interact with the N4 outer membrane receptor, NfrA^14^. phiAxp-2 ORF28 encodes a capsid and scaffold protein that is absent in AH2. The scaffold protein assists in the assembly of the outer shell and dissociates from the capsid during subsequent DNA packaging^11^. ORF29 encodes a tail assembly protein that has 31% amino acid identity with the AH2 tail assembly protein. ORF33 and ORF34 encode a tail assembly protein and a virion-associated protein, respectively. Neither is present in AH2. Following the structural components, there is a region encoding small and uncharacterized proteins, which spans about 24 kb. No genes similar to the genes for lysin or holin have yet been detected in the phiAxp-2 genome, which are responsible for host cell disruption during the burst steps of phages^15^, although the clearing of the bacterial culture at a specific time point strongly suggest that they exist.

### Identification of the host receptor

**Figure 7:**
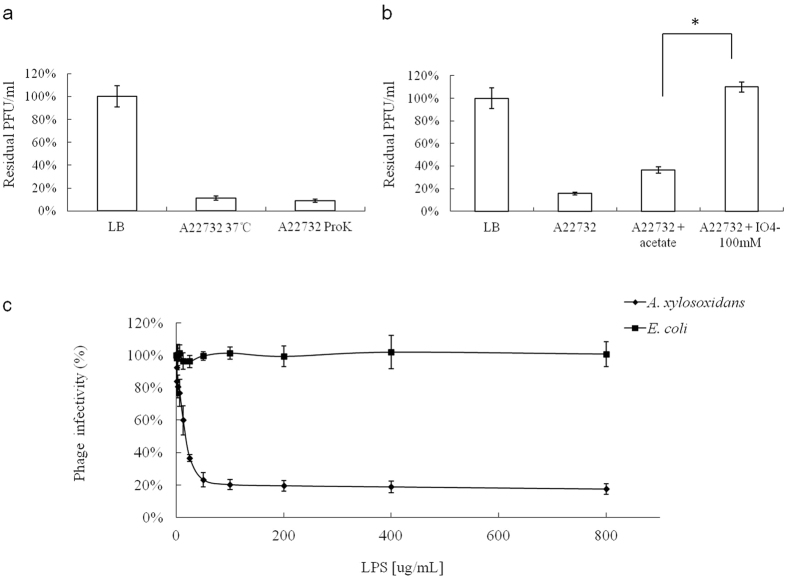
Effects of different treatments applied to the host bacterium on phiAxp-2 adsorption, which is shown as residual pfu percentages. (**a**) Effect of proteinase K treatment on the adsorption of phiAxp-2 to *A. xylosoxidans* strain A22732. (**b**) Effect of periodate treatment on the adsorption of phiAxp-2 to *A. xylosoxidans* strain A22732. The controls (LB and “A22732+ acetate”), untreated (A22732), and treated groups (“A22732+ ProtK”, treated with proteinase K; “A22732+ IO^4−^”, treated with periodate) were tested for adsorption, as indicated on the *x* axes. Error bars denote statistical variations. Significance was determined with one-sample Student’s *t* test when the treated and untreated groups were compared. ^*^P < 0.05. (**c**) Inactivation of phiAxp-2 by LPS derived from *A. xylosoxidans* A22732. Percentage infectivity was determined after incubation for 1 h at 37 °C. Error bars denote statistical variations.

The adsorption of the phage to the bacterial surface is the first and most important step in the phage infection process. Both the lipopolysaccharide (LPS) and outer membrane proteins located on the surfaces of Gram-negative bacteria can be used as phage receptors. In the present study, protease K and periodate were used to destroy the *A. xylosoxidans* outer membrane proteins and LPS, respectively, to determine the attachment site for phage phiAxp-2 on the cell surface of *A. xylosoxidans* (Fig. 7a,b). Phage adsorption to LPS-deficient *A. xylosoxidans* cells was inhibited, indicating that phage-specific adhesion is mediated by LPS (Fig. 7b). These results were confirmed with a phage inactivation assay performed with pure LPS isolated from strain A22732. These experiments showed a direct correlation between the LPS concentration and the inhibition of viral particle infectivity (Fig. 7c), and approximately 12.5 μg/ml LPS inhibited the activity of 50% of 2.8 × 10^3^ pfu phiAxp-2. LPS of *E. coli* 0111:B4 was used as the negative control and showed no phage-inactivating capacity compared with *A. xylosoxidans* LPS, indicating that *A. xylosoxidans* LPS is the specific receptor for phage phiAxp-2.

### Concluding remarks

In this study, we have characterized a *Siphoviridae* phage that infects the important nosocomial pathogen *A. xylosoxidans*. Genomic data are an important resource with which to study and engineer phages to control specific bacterial species^15^, and advances in phage genomic characterization have made phage therapy more feasible in terms of both its logistics and safety^10^. A combination of genomic sequencing and a morphological analysis showed that phiAxp-2 is a member of the family *Siphoviridae*, and is related to the previously sequenced phage AH2. The most striking feature to emerge from a comparative analysis of the phage genome was the extensive mosaic structure of phiAxp-2, which contains different segments with distinct evolutionary histories. The results of this comparative analysis indicate that the left half of the phiAxp-2 genome has a similar genomic structure to partial genomic sequences of AH2. A simple general explanation is that horizontal genetic exchange has played a dominant role in shaping these genomic architectures. Gene modules are exchanged using host- or phage-encoded recombination machinery^16^. This analysis provides an important contribution to the field of bacteriophage genomics and a foundation upon which to extend our understanding of the natural predecessors of *A. xylosoxidans*. Further clarification of the functions of the unique hypothetical phage proteins identified may provide new insight into the mechanisms of genome evolution. Identification of the receptor molecules of phages provides crucial insight into the early stages of infection^17^. Our results show that phage phiAxp-2 recognizes LPS as its primary receptor for adsorption. Further studies of this phage will be useful in understanding the role of phages in evolution and bacterial lifestyles.

## Methods

### Bacterial strains, bacteriophage, and media

A 16S rDNA sequence analysis was used to identify the host bacterium. The multidrug-resistant *A. xylosoxidans* A22732 strain was used as an indicator for phage screening of sewage samples. The samples were centrifuged at 12,000 × g for 10 min to remove the solid impurities, the supernatants were filtered through a 0.22 μm pore-size membrane filter to remove bacterial debris. The filtrates were mixed with *A. xylosoxidans* culture to enrich the phage at 37 °C. Following enrichment, the culture was centrifuged at 10,000 × g for 10 min, and then the supernatant was filtered with a 0.22 μm pore-size membrane filter to remove the residual bacterial cells. The filtrate (0.1 ml) was mixed with 0.3 ml of *A. xylosoxidans* in LB culture and 3 ml of molten top soft nutrient agar (0.75% agar), which was then overlaid onto solidified base nutrient agar (1.5% agar). Following incubation for 6 h, clear phage plaques were picked from the plate^18^. The phage titer was determined with the double-layered method. Luria–Bertani (LB) broth or LB agar was used to culture the bacterium. The host range of the phage was tested against 17 clinical strains from our microorganism center with standard spot tests^19^.

### Electron microscopy

Phage particles were allowed to adsorb onto a carbon-coated copper grid before they were negatively stained with 2% phosphotungstic acid (pH 7.0). After the grid was dried at room temperature, it was examined under a Philips TECNAI-10 transmission electron microscope.

### One-step growth curve

For the one-step growth curve analysis, *A. xylosoxidans* cells (optical density at 600 nm [OD_600_] = 0.5) were infected with phage phiAxp-2 at a multiplicity of infection of 0.0001. The bacteriophage was allowed to adsorb for 5 min at 37 °C. The mixture was then centrifuged at 12,000 × g for 30 s to remove any unadsorbed phage particles, and the resultant pellet was resuspended in 5 ml of LB medium. The samples were incubated at 37 °C and collected every 20 min for 300 min.

### Stability studies

A temperature-controlled incubator was used to determine the stability of phiAxp-2 at different pHs or temperatures, and in the presence of disinfectants or ions. Briefly, a 1.5 ml tube containing a filter-sterilized phage sample was incubated at a specified temperature or pH. After the desired treatment, the tube was cooled slowly and placed in an ice-water bath, and the samples were assayed to determine the surviving plaque-forming units (pfu). To test the phage sensitivity to disinfectants and ions, a known amount of phage was incubated with different concentrations of disinfectants or ions. The plates were then overlain with liquefied soft agar (LB with 0.75% [w/v] agar) containing the host cells and incubated at 37 °C for 12 h.

### Genome sequencing and assembly

The purified phage DNA was sequenced with an Illumina HiSeq 2500 sequencer. The sequence reads were filtered to remove low-quality sequences and trimmed to remove adaptor sequences, and the filtered sequences were assembled. The final assembled sequence was compared with current protein and nucleotide databases (http://www.ncbi.nlm.nih.gov/) using the Basic Local Alignment Search Tool (Blast) software^20^. BlastP was used to determine the similarity of the deduced phage proteins to proteins in the National Center for Biotechnology Information (NCBI) database (http://www.ncbi.nlm.nih.gov). Simulation of the restriction enzyme mapping of the phiAxp-2 genome sequence was carried out using the software package DNAStar. The phiAxp-2 DNA was digested by selected restriction endonucleases (*HindIII*, purchased from New England Biolabs, Ipswich, MA, USA). For a reaction system of 20 μL, 10 units of the restriction endonuclease and 300 ng of phiAxp-2 DNA were used. The mixture was incubated at 37 °C for 180 min and then used to perform agarose gel electrophoresis. Agarose gel electrophoresis was subsequently performed to separate the restriction fragments. The CLC Main Workbench, version 6.1.1 (CLC bio, Aarhus, Denmark) was used to annotate the genome. The computer-based predictions were checked manually. A phylogenetic analysis, together with the published genomic sequences of related phages, was conducted with ClustalW (Slow/Accurate, IUB). Whole-genome comparisons were made with Mauve^21^.

### Identification of the phage receptor

The receptor properties of phiAxp-2 were determined as described previously^22^. Briefly, *A. xylosoxidans* A22732 cultures were treated with sodium acetate (50 mM, pH 5.2) containing 100 mM IO^4−^ at room temperature for 2 h (protected from light) or proteinase K (0.2 mg/ml; Promega) at 37 °C for 3 h to determine whether proteinase K or periodate destroys the phage receptor. A phage adsorption assay was then performed, as previously described^23^. LB medium was used as the nonadsorbing control in each assay, and the phage titer in the control supernatant was set to 100%. Each assay was performed in duplicate and repeated twice^22^.

### Phage inactivation by LPS

LPS was extracted from *A. xylosoxidans* using an LPS extraction kit from Intron Biotechnology (17144; Boca Scientific, Boca Raton, FL, USA), according to the manufacturer’s instructions. A control containing LPS from *Escherichia coli* O111:B4, purchased from Sigma-Aldrich, Inc. (L2630; Sigma, USA), was used as the negative control to ensure that any effect was specific to *A. xylosoxidans* LPS. Phage inactivation by LPS was tested as previously described^24^.

### Nucleotide sequence accession number

The nucleotide sequence of phiAxp-2 phage reported in this article has been deposited in the GenBank database as accession number KT321316.

## Additional Information

**How to cite this article**: Li, E. *et al*. Identification and molecular characterization of bacteriophage phiAxp-2 of *Achromobacter xylosoxidans*. *Sci. Rep.* **6**, 34300; doi: 10.1038/srep34300 (2016).

## Figures and Tables

**Table 1:** Host range analysis of the phage phiAxp-2 −, absent; +, present.

Species	ID	Infection
*Achromobacter xylosoxidans*	A22732	+
*A. xylosoxidans*	5271	+
*A. xylosoxidans*	844	+
*A. xylosoxidans*	6065	+
*Escherichia coli*	ATCC 25922	−
*Klebsiella pneumoniae*	ATCC BAA-1706	−
*Serratia marcescens*	wk2050	−
*Enterobacter aerogenes*	3-SP	−
*Enterobacter cloacae*	T5282	−
*Leclercia adcarboxglata*	P10164	−
*Raoultella ornithinolytica*	YNKP001	−
*Stenotrophomonas maltophilia*	9665	−
*Citrobacter freundii*	P10159	−
*Vibrio parahaemolyticus*	J5421	−
*Pseudomonas aeruginosa*	PA01	−
*Acinetobacter baumannii*	N1	−
*Shigella sonnei*	#1083	−

**Table 2:** *Achromobacter* phage phiAxp-2 gene annotations.

ORFs	Strand	Start	End	Length (aa)^a^	aa identity (%)	Function
orf01	−	444	2966	840	42	DNA primase [*Burkholderia* phage AH2]
orf02	−	3002	3301	99	—	—
orf03	+	3451	3957	168	37	hypothetical protein Nazgul22 [*Burkholderia* phage BcepNazgul]
orf04	+	4042	4437	131	—	—
orf05	+	4453	5745	430	38	exonuclease [*Burkholderia* phage AH2]
orf06	+	5792	6430	212	35	single-stranded DNA binding protein [*Burkholderia* phage AH2]
orf07	+	6507	8600	697	43	DNA polymerase [*Burkholderia* phage AH2]
orf08	+	8597	8890	97	38	resolvase [*Burkholderia* phage AH2]
orf09	+	8937	10481	514	45	helicase [*Burkholderia* phage AH2]
orf10	+	10528	10737	69	57	hypothetical protein Q051_01461 [*Pseudomonas aeruginosa* BWHPSA046]
orf11	+	10727	11326	199	32	terminase small subunit [*Burkholderia* phage AH2]
orf12	+	11313	13400	694	54	terminase large subunit [*Burkholderia* phage AH2]
orf13	+	13400	13651	83	39	head-tail joining protein [*Burkholderia* phage AH2]
orf14	+	13682	15292	536	57	portal protein [*Burkholderia* phage AH2]
orf15	+	15296	16675	459	42	prohead protease [*Burkholderia* phage AH2]
orf16	+	16703	17080	125	29	decorator protein [*Burkholderia* phage AH2]
orf17	+	17108	18163	351	47	major capsid protein [*Burkholderia* phage AH2]
orf18	+	18233	18523	96	38	hypothetical protein [*Pseudomonas aeruginosa*]
orf19	+	18550	18783	77	—	—
orf20	+	18783	19205	140	32	hypothetical protein AH2_00060 [*Burkholderia* phage AH2]
orf21	+	19208	19834	208	55	hypothetical protein AH2_00059 [*Burkholderia* phage AH2]
orf22	+	19827	20420	197	34	minor tail protein [*Burkholderia* phage AH2]
orf23	+	20456	21253	265	50	hypothetical protein AH2_00057 [*Burkholderia* phage AH2]
orf24	+	21263	21754	163	31	tail protein [*Burkholderia* phage AH2]
orf25	+	21757	21966	69	43	tail protein [*Burkholderia* phage AH2]
orf26	+	21950	26752	1603	33	tape measure protein [*Bradyrhizobium* sp. WSM3983]
orf27	+	26749	30924	1387	42	tail sheath [*Escherichia* phage N4]
orf28	+	30927	33446	839	51	capsid and scaffold protein [*Delftia* phage RG-2014]
orf29	+	33448	34290	280	31	tail assembly protein [*Burkholderia* phage AH2]
orf30	+	34301	34426	41	—	—
orf31	+	34414	34653	79	—	—
orf32	+	34686	34877	63	—	—
orf33	+	34874	37261	795	44	tail assembly protein [*Bradyrhizobium* sp. WSM3983]
orf34	+	37258	38034	258	36	virion associated protein [*Xylella* phage Salvo]
orf35	+	38046	38780	244	—	—
orf36	+	38850	39266	138	—	—
orf37	+	39424	39699	91	—	—
orf38	+	39996	40481	161	—	—
orf39	+	40536	40745	87	—	—
orf40	+	40729	40887	52	—	—
orf41	−	40934	41860	308	—	—
orf42	−	41857	42147	96	—	—
orf43	−	42149	42520	123	—	—
orf44	−	42510	42731	73	—	—
orf45	−	42728	42982	84	—	—
orf46	−	42979	43158	59	—	—
orf47	−	43179	43490	103	—	—
orf48	−	43480	43917	145	—	—
orf49	−	44030	44233	67	—	—
orf50	−	44230	44595	121	—	—
orf51	−	44598	45155	185	—	—
orf52	−	45216	45710	164	—	—
orf53	−	45764	46351	195	—	—
orf54	−	46353	46538	61	—	—
orf55	−	46816	47238	140	—	—
orf56	−	47302	47901	199	—	—
orf57	−	47903	48052	49	—	—
orf58	−	48052	48309	85	—	—
orf59	−	48309	48671	120	—	—
orf60	−	48674	48946	90	—	—
orf61	−	48943	49611	222	—	—
orf62	−	49608	49889	93	—	—
orf63	−	49876	51153	425	—	—
orf64	−	51179	51472	97	—	—
orf65	−	51474	51764	96	—	—
orf66	−	51860	52297	145	—	—
orf67	+	52792	53001	69	—	—
orf68	+	53072	54028	318	—	—
orf69	+	54041	54736	231	—	—
orf70	+	54761	55336	191	—	—
orf71	+	55333	55644	103	—	—
orf72	+	55858	56799	313	—	—
orf73	+	56802	57029	80	—	—
orf74	+	57026	57148	40	—	—
orf75	+	57153	57476	107	—	—
orf76	+	57481	57762	93	—	—
orf77	+	57988	58239	109	—	—
orf78	+	58236	58466	76	—	—
orf79	+	58480	58920	146	—	—
orf80	+	59004	59153	50	—	—
orf81	+	59168	59296	42	—	—
orf82	+	59301	59789	162	—	—
orf83	+	59808	60353	181	—	—
orf84	+	60358	60711	117	—	—
orf85	+	60720	61406	228	—	—
orf86	+	61465	61638	57	—	—

^a^Amino acids.
